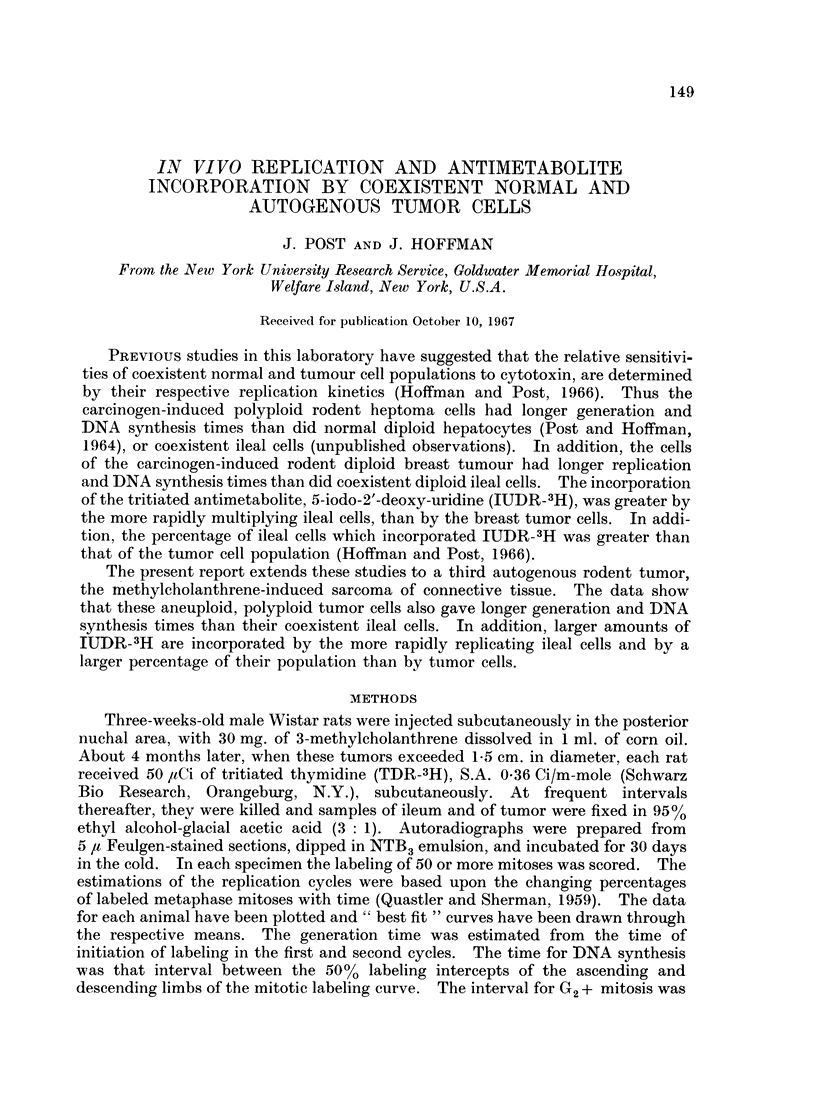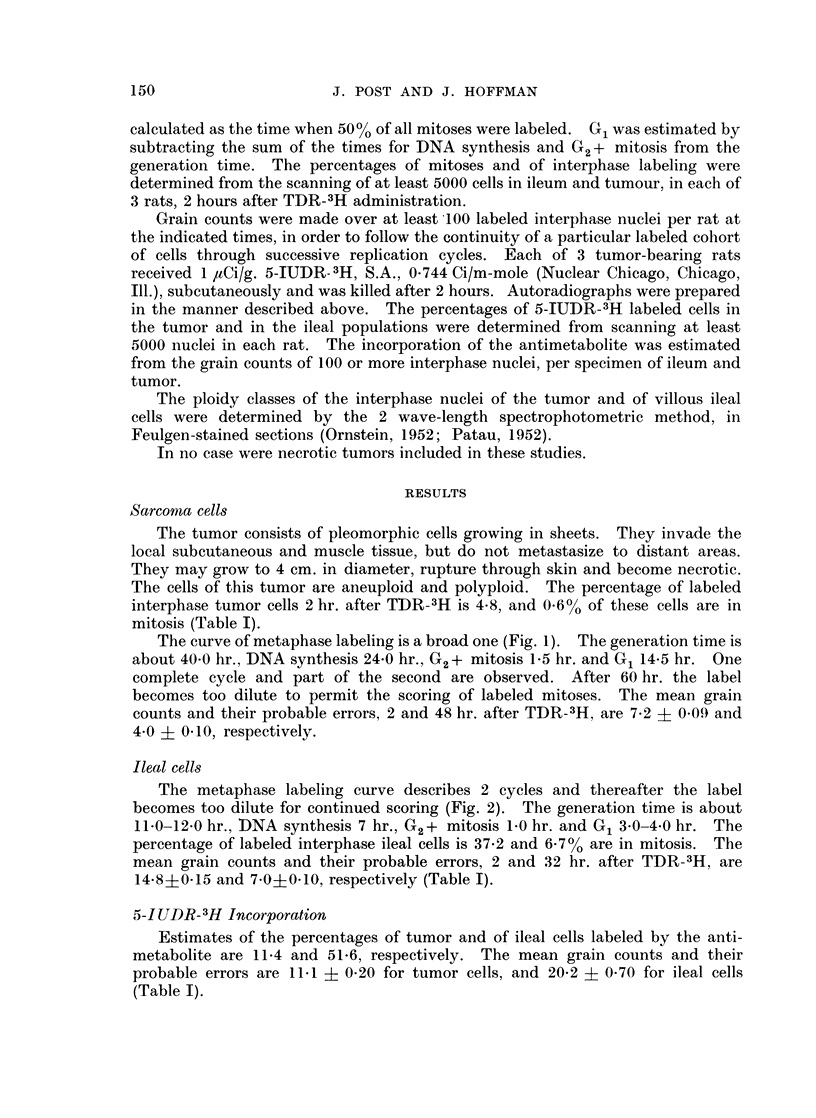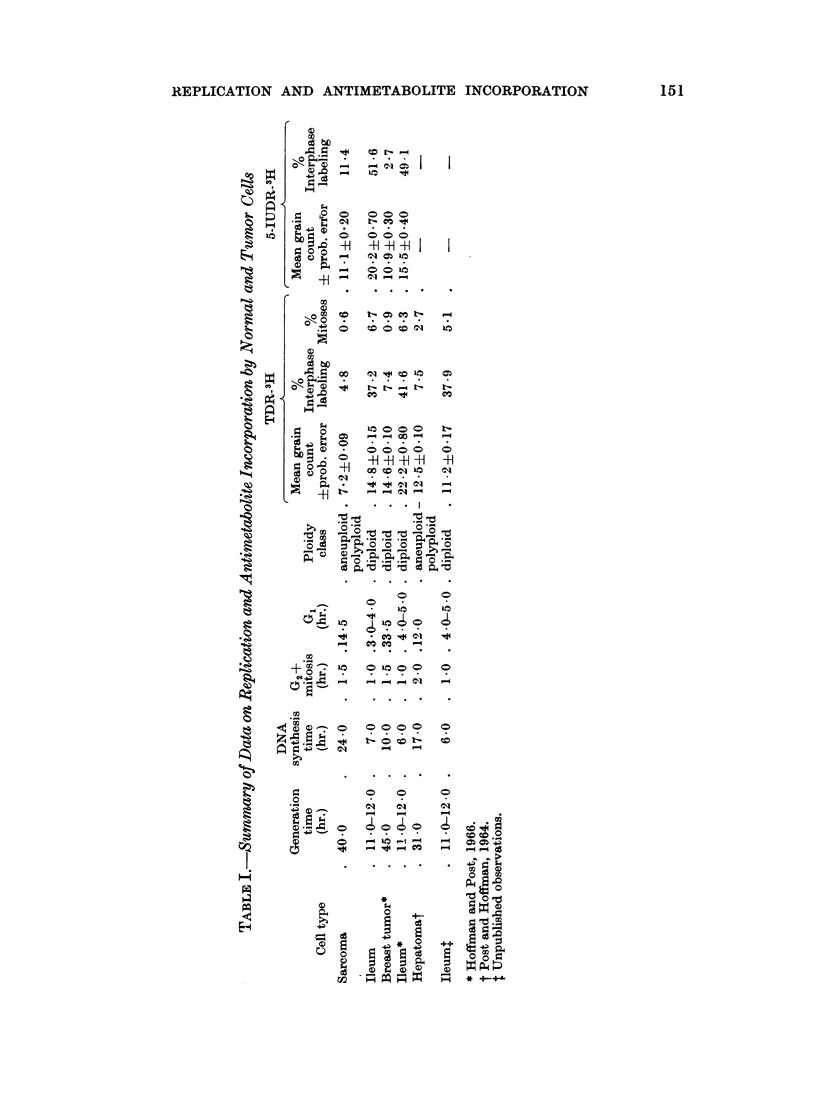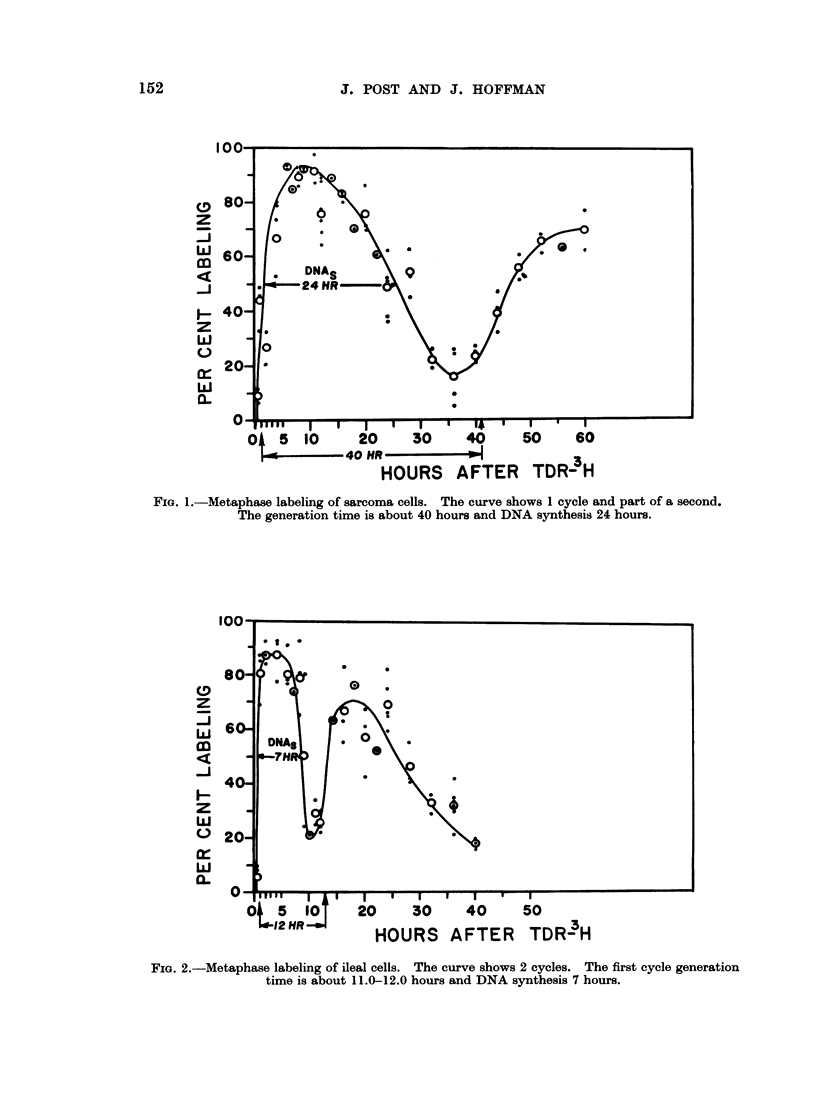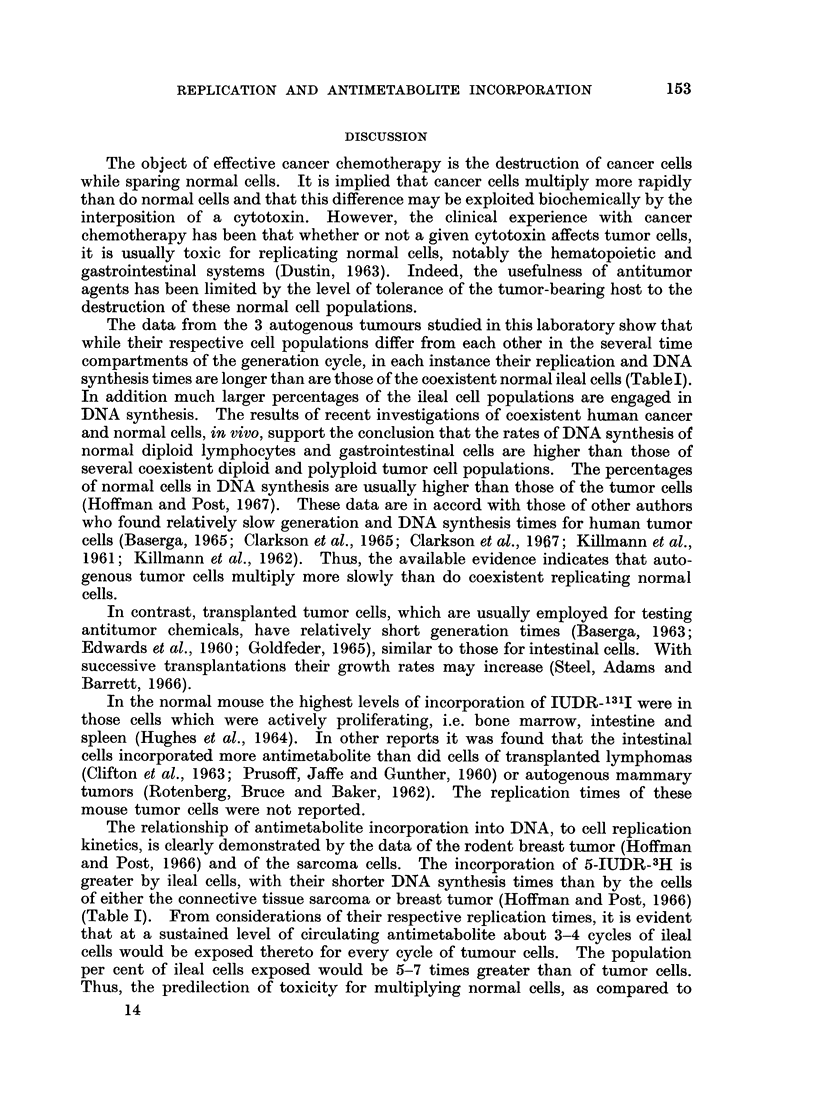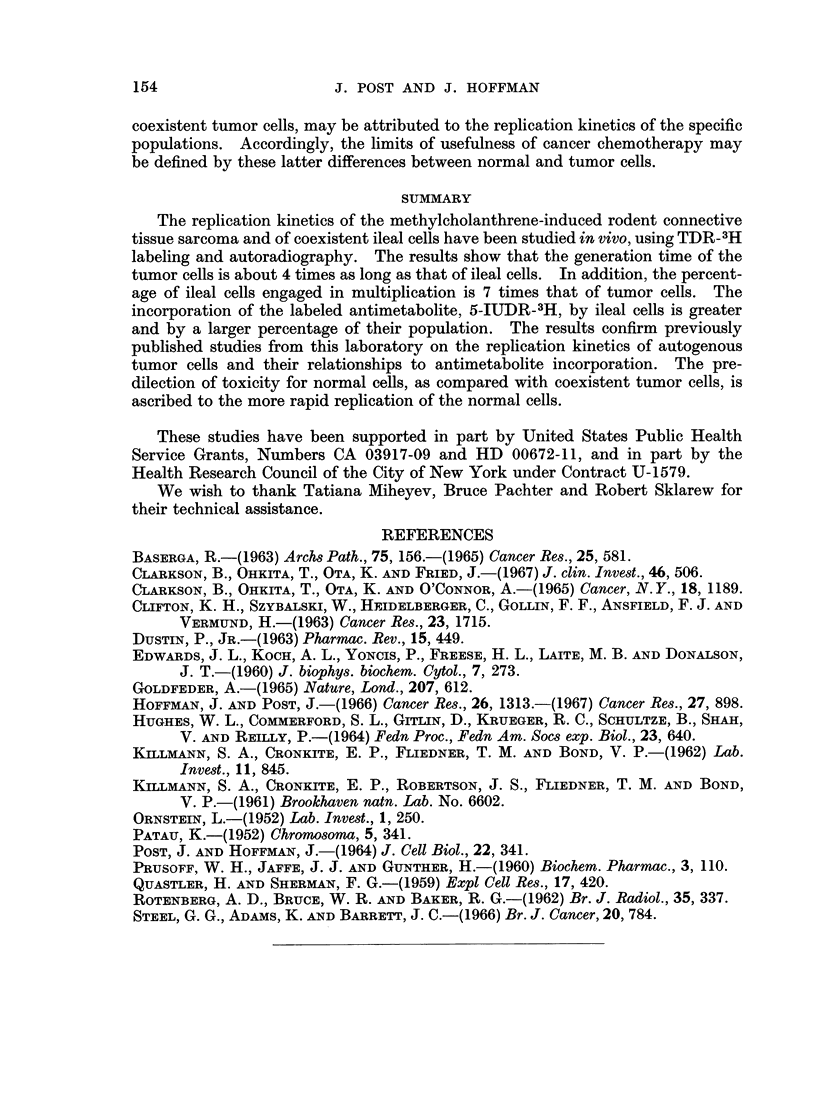# In vio replication and antimetabolite incorporation by coexistent normal and autogenous tumor cells.

**DOI:** 10.1038/bjc.1968.21

**Published:** 1968-03

**Authors:** J. Post, J. Hoffman


### *IN VIVO* REPLICATION AND ANTIMETABOLITE INCORPORATION BY COEXISTENT NORMAL AND AUTOGENOUS TUMOR CELLS

J. POST AND J. HOFFMAN

*From the New York University Research Service, Goldwater Memorial Hospital, Welfare Island, New York, U.S.A.*

Received for publication October 10, 1967

PREVIOUS studies in this laboratory have suggested that the relative sensitivities of coexistent normal and tumour cell populations to cytotoxin, are determined by their respective replication kinetics (Hoffman and Post, 1966). Thus the carcinogen-induced polyploid rodent heptoma cells had longer generation and DNA synthesis times than did normal diploid hepatocytes (Post and Hoffman, 1964), or coexistent ileal cells (unpublished observations). In addition, the cells of the carcinogen-induced rodent diploid breast tumour had longer replication and DNA synthesis times than did coexistent diploid ileal cells. The incorporation of the tritiated antimetabolite, 5-iodo-2′-deoxy-uridine (IUDR-$^3$H), was greater by the more rapidly multiplying ileal cells, than by the breast tumor cells. In addition, the percentage of ileal cells which incorporated IUDR-$^3$H was greater than that of the tumor cell population (Hoffman and Post, 1966).

The present report extends these studies to a third autogenous rodent tumor, the methylcholanthrene-induced sarcoma of connective tissue. The data show that these aneuploid, polyploid tumor cells also gave longer generation and DNA synthesis times than their coexistent ileal cells. In addition, larger amounts of IUDR-$^3$H are incorporated by the more rapidly replicating ileal cells and by a larger percentage of their population than by tumor cells.

### METHODS

Three-weeks-old male Wistar rats were injected subcutaneously in the posterior nuchal area, with 30 mg. of 3-methylcholanthrene dissolved in 1 ml. of corn oil. About 4 months later, when these tumors exceeded 1·5 cm. in diameter, each rat received 50 $\mu$Ci of tritiated thymidine (TDR-$^3$H), S.A. 0·36 Ci/m-mole (Schwarz Bio Research, Orangeburg, N.Y.), subcutaneously. At frequent intervals thereafter, they were killed and samples of ileum and of tumor were fixed in 95% ethyl alcohol-glacial acetic acid (3 : 1). Autoradiographs were prepared from 5 $\mu$ Feulgen-stained sections, dipped in $NTB_3$ emulsion, and incubated for 30 days in the cold. In each specimen the labeling of 50 or more mitoses was scored. The estimations of the replication cycles were based upon the changing percentages of labeled metaphase mitoses with time (Quastler and Sherman, 1959). The data for each animal have been plotted and " best fit " curves have been drawn through the respective means. The generation time was estimated from the time of initiation of labeling in the first and second cycles. The time for DNA synthesis was that interval between the 50% labeling intercepts of the ascending and descending limbs of the mitotic labeling curve. The interval for $G_2+$ mitosis was calculated as the time when 50% of all mitoses were labeled. $G_1$ was estimated by subtracting the sum of the times for DNA synthesis and $G_2+$ mitosis from the generation time. The percentages of mitoses and of interphase labeling were determined from the scanning of at least 5000 cells in ileum and tumour, in each of 3 rats, 2 hours after TDR-$^3$H administration.

Grain counts were made over at least 100 labeled interphase nuclei per rat at the indicated times, in order to follow the continuity of a particular labeled cohort of cells through successive replication cycles. Each of 3 tumor-bearing rats received 1 $\mu$Ci/g. 5-IUDR-$^3$H, S.A., 0·744 Ci/m-mole (Nuclear Chicago, Chicago, Ill.), subcutaneously and was killed after 2 hours. Autoradiographs were prepared in the manner described above. The percentages of 5-IUDR-$^3$H labeled cells in the tumor and in the ileal populations were determined from scanning at least 5000 nuclei in each rat. The incorporation of the antimetabolite was estimated from the grain counts of 100 or more interphase nuclei, per specimen of ileum and tumor.

The ploidy classes of the interphase nuclei of the tumor and of villous ileal cells were determined by the 2 wave-length spectrophotometric method, in Feulgen-stained sections (Ornstein, 1952; Patau, 1952).

In no case were necrotic tumors included in these studies.

### RESULTS

### *Sarcoma cells*

The tumor consists of pleomorphic cells growing in sheets. They invade the local subcutaneous and muscle tissue, but do not metastasize to distant areas. They may grow to 4 cm. in diameter, rupture through skin and become necrotic. The cells of this tumor are aneuploid and polyploid. The percentage of labeled interphase tumor cells 2 hr. after TDR-$^3$H is 4·8, and 0·6% of these cells are in mitosis (Table I).

The curve of metaphase labeling is a broad one (Fig. 1). The generation time is about 40·0 hr., DNA synthesis 24·0 hr., $G_2+$ mitosis 1·5 hr. and $G_1$ 14·5 hr. One complete cycle and part of the second are observed. After 60 hr. the label becomes too dilute to permit the scoring of labeled mitoses. The mean grain counts and their probable errors, 2 and 48 hr. after TDR-$^3$H, are $7·2 \pm 0·09$ and $4·0 \pm 0·10$, respectively.

### *Ileal cells*

The metaphase labeling curve describes 2 cycles and thereafter the label becomes too dilute for continued scoring (Fig. 2). The generation time is about 11·0–12·0 hr., DNA synthesis 7 hr., $G_2+$ mitosis 1·0 hr. and $G_1$ 3·0–4·0 hr. The percentage of labeled interphase ileal cells is 37·2 and 6·7% are in mitosis. The mean grain counts and their probable errors, 2 and 32 hr. after TDR-$^3$H, are $14·8 \pm 0·15$ and $7·0 \pm 0·10$, respectively (Table I).

### *5-IUDR-$^3$H Incorporation*

Estimates of the percentages of tumor and of ileal cells labeled by the antimetabolite are 11·4 and 51·6, respectively. The mean grain counts and their probable errors are $11·1 \pm 0·20$ for tumor cells, and $20·2 \pm 0·70$ for ileal cells (Table I).

TABLE I.—*Summary of Data on Replication and Antimetabolite Incorporation by Normal and Tumor Cells*

| Cell type | Generation time (hr.) | DNA synthesis time (hr.) | $G_2$+ mitosis (hr.) | $G_1$ (hr.) | Ploidy class | TDR-$^3$H: Mean grain count ±prob. error | TDR-$^3$H: % Interphase labeling | TDR-$^3$H: % Mitoses | 5-IUDR-$^3$H: Mean grain count ± prob. error | 5-IUDR-$^3$H: % Interphase labeling |
|---|---|---|---|---|---|---|---|---|---|---|
| Sarcoma | 40·0 | 24·0 | 1·5 | 14·5 | aneuploid polyploid | 7·2±0·09 | 4·8 | 0·6 | 11·1±0·20 | 11·4 |
| Ileum | 11·0–12·0 | 7·0 | 1·0 | 3·0–4·0 | diploid | 14·8±0·15 | 37·2 | 6·7 | 20·2±0·70 | 51·6 |
| Breast tumor* | 45·0 | 10·0 | 1·5 | 33·5 | diploid | 14·6±0·10 | 7·4 | 0·9 | 10·9±0·30 | 2·7 |
| Ileum* | 11·0–12·0 | 6·0 | 1·0 | 4·0–5·0 | diploid | 22·2±0·80 | 41·6 | 6·3 | 15·5±0·40 | 49·1 |
| Hepatoma† | 31·0 | 17·0 | 2·0 | 12·0 | aneuploid–polyploid | 12·5±0·10 | 7·5 | 2·7 | — | — |
| Ileum‡ | 11·0–12·0 | 6·0 | 1·0 | 4·0–5·0 | diploid | 11·2±0·17 | 37·9 | 5·1 | — | — |

* Hoffman and Post, 1966.

† Post and Hoffman, 1964.

‡ Unpublished observations.

FIG. 1.—Metaphase labeling of sarcoma cells. The curve shows 1 cycle and part of a second. The generation time is about 40 hours and DNA synthesis 24 hours.

FIG. 2.—Metaphase labeling of ileal cells. The curve shows 2 cycles. The first cycle generation time is about 11.0–12.0 hours and DNA synthesis 7 hours.

DISCUSSION

The object of effective cancer chemotherapy is the destruction of cancer cells while sparing normal cells. It is implied that cancer cells multiply more rapidly than do normal cells and that this difference may be exploited biochemically by the interposition of a cytotoxin. However, the clinical experience with cancer chemotherapy has been that whether or not a given cytotoxin affects tumor cells, it is usually toxic for replicating normal cells, notably the hematopoietic and gastrointestinal systems (Dustin, 1963). Indeed, the usefulness of antitumor agents has been limited by the level of tolerance of the tumor-bearing host to the destruction of these normal cell populations.

The data from the 3 autogenous tumours studied in this laboratory show that while their respective cell populations differ from each other in the several time compartments of the generation cycle, in each instance their replication and DNA synthesis times are longer than are those of the coexistent normal ileal cells (Table I). In addition much larger percentages of the ileal cell populations are engaged in DNA synthesis. The results of recent investigations of coexistent human cancer and normal cells, *in vivo,* support the conclusion that the rates of DNA synthesis of normal diploid lymphocytes and gastrointestinal cells are higher than those of several coexistent diploid and polyploid tumor cell populations. The percentages of normal cells in DNA synthesis are usually higher than those of the tumor cells (Hoffman and Post, 1967). These data are in accord with those of other authors who found relatively slow generation and DNA synthesis times for human tumor cells (Baserga, 1965; Clarkson *et al.*, 1965; Clarkson *et al.*, 1967; Killmann *et al.*, 1961; Killmann *et al.*, 1962). Thus, the available evidence indicates that autogenous tumor cells multiply more slowly than do coexistent replicating normal cells.

In contrast, transplanted tumor cells, which are usually employed for testing antitumor chemicals, have relatively short generation times (Baserga, 1963; Edwards *et al.*, 1960; Goldfeder, 1965), similar to those for intestinal cells. With successive transplantations their growth rates may increase (Steel, Adams and Barrett, 1966).

In the normal mouse the highest levels of incorporation of IUDR-$^{131}$I were in those cells which were actively proliferating, i.e. bone marrow, intestine and spleen (Hughes *et al.*, 1964). In other reports it was found that the intestinal cells incorporated more antimetabolite than did cells of transplanted lymphomas (Clifton *et al.*, 1963; Prusoff, Jaffe and Gunther, 1960) or autogenous mammary tumors (Rotenberg, Bruce and Baker, 1962). The replication times of these mouse tumor cells were not reported.

The relationship of antimetabolite incorporation into DNA, to cell replication kinetics, is clearly demonstrated by the data of the rodent breast tumor (Hoffman and Post, 1966) and of the sarcoma cells. The incorporation of 5-IUDR-$^{3}$H is greater by ileal cells, with their shorter DNA synthesis times than by the cells of either the connective tissue sarcoma or breast tumor (Hoffman and Post, 1966) (Table I). From considerations of their respective replication times, it is evident that at a sustained level of circulating antimetabolite about 3–4 cycles of ileal cells would be exposed thereto for every cycle of tumour cells. The population per cent of ileal cells exposed would be 5–7 times greater than of tumor cells. Thus, the predilection of toxicity for multiplying normal cells, as compared to coexistent tumor cells, may be attributed to the replication kinetics of the specific populations. Accordingly, the limits of usefulness of cancer chemotherapy may be defined by these latter differences between normal and tumor cells.

SUMMARY

The replication kinetics of the methylcholanthrene-induced rodent connective tissue sarcoma and of coexistent ileal cells have been studied *in vivo*, using TDR-$^3$H labeling and autoradiography. The results show that the generation time of the tumor cells is about 4 times as long as that of ileal cells. In addition, the percentage of ileal cells engaged in multiplication is 7 times that of tumor cells. The incorporation of the labeled antimetabolite, 5-IUDR-$^3$H, by ileal cells is greater and by a larger percentage of their population. The results confirm previously published studies from this laboratory on the replication kinetics of autogenous tumor cells and their relationships to antimetabolite incorporation. The predilection of toxicity for normal cells, as compared with coexistent tumor cells, is ascribed to the more rapid replication of the normal cells.

These studies have been supported in part by United States Public Health Service Grants, Numbers CA 03917-09 and HD 00672-11, and in part by the Health Research Council of the City of New York under Contract U-1579.

We wish to thank Tatiana Miheyev, Bruce Pachter and Robert Sklarew for their technical assistance.

REFERENCES

Baserga, R.—(1963) *Archs Path.*, **75**, 156.—(1965) *Cancer Res.*, **25**, 581.
Clarkson, B., Ohkita, T., Ota, K. and Fried, J.—(1967) *J. clin. Invest.*, **46**, 506.
Clarkson, B., Ohkita, T., Ota, K. and O'Connor, A.—(1965) *Cancer, N.Y.*, **18**, 1189.
Clifton, K. H., Szybalski, W., Heidelberger, C., Gollin, F. F., Ansfield, F. J. and Vermund, H.—(1963) *Cancer Res.*, **23**, 1715.
Dustin, P., Jr.—(1963) *Pharmac. Rev.*, **15**, 449.
Edwards, J. L., Koch, A. L., Yoncis, P., Freese, H. L., Laite, M. B. and Donalson, J. T.—(1960) *J. biophys. biochem. Cytol.*, **7**, 273.
Goldfeder, A.—(1965) *Nature, Lond.*, **207**, 612.
Hoffman, J. and Post, J.—(1966) *Cancer Res.*, **26**, 1313.—(1967) *Cancer Res.*, **27**, 898.
Hughes, W. L., Commerford, S. L., Gitlin, D., Krueger, R. C., Schultze, B., Shah, V. and Reilly, P.—(1964) *Fedn Proc., Fedn Am. Socs exp. Biol.*, **23**, 640.
Killmann, S. A., Cronkite, E. P., Fliedner, T. M. and Bond, V. P.—(1962) *Lab. Invest.*, **11**, 845.
Killmann, S. A., Cronkite, E. P., Robertson, J. S., Fliedner, T. M. and Bond, V. P.—(1961) *Brookhaven natn. Lab.* No. 6602.
Ornstein, L.—(1952) *Lab. Invest.*, **1**, 250.
Patau, K.—(1952) *Chromosoma*, **5**, 341.
Post, J. and Hoffman, J.—(1964) *J. Cell Biol.*, **22**, 341.
Prusoff, W. H., Jaffe, J. J. and Gunther, H.—(1960) *Biochem. Pharmac.*, **3**, 110.
Quastler, H. and Sherman, F. G.—(1959) *Expl Cell Res.*, **17**, 420.
Rotenberg, A. D., Bruce, W. R. and Baker, R. G.—(1962) *Br. J. Radiol.*, **35**, 337.
Steel, G. G., Adams, K. and Barrett, J. C.—(1966) *Br. J. Cancer*, **20**, 784.